# The Dynamics of the Gender Gap at Retirement in Italy: Evidence from SHARE

**DOI:** 10.1007/s40797-022-00201-7

**Published:** 2022-07-22

**Authors:** Antonio Abatemarco, Maria Russolillo

**Affiliations:** 1 Department of Economics and Statistics and CELPE (Centre for Economic and Labour Policy Evaluation), University of Salerno, Via Giovanni Paolo II, 132, 84084 Fisciano, SA Italy; 2 Department of Economics and Statistics, University of Salerno, Via Giovanni Paolo II, 132, 84084 Fisciano, SA Italy; 3 NetSPAR (Network for Studies on Pensions, Aging and Retirement), Tilburg University, Tilburg, The Netherlands

**Keywords:** Gender gap, Pension, Redistribution, Actuarial fairness, H55, J16, J26

## Abstract

We investigate the dynamics of the gender gap at retirement in Italy—by cohort and by year of retirement—for individuals retiring from 1980 to 2027 using data from SHARELIFE (wave 3 and wave 7). Most importantly, we disentangle the opposite effects on the gender gap originating respectively from (i) improving labor market conditions for women, and (ii) increasing actuarial fairness of the pension plan due to the progressive transition from a defined-benefit to a notional defined-contribution scheme. To capture the impact of these two driving forces, we implement a counterfactual analysis by which the gender gap at retirement is measured both in the actual and in the virtual distribution of pension benefits, with the latter being obtained under the hypothesis of an actuarially fair pension scheme. We observe a U-shaped pattern since the actual gender gap at retirement is found to be decreasing up to 2016 but increasing after this date. Specifically, the increasing pattern for the gender gap at retirement after 2016 is shown to be driven by (i) decreasing redistributive impact of the pension scheme, and (ii) women’s penalization in the pro-rata mechanism due to lower contributions paid in the early working life.

## Introduction

Gender inequality at retirement is known to originate from the gender gap in the labor market—when pension savings are accumulated—and to be affected by the design of the pension scheme determining the first pension benefit at retirement. Both these determinants have sensibly changed in the last decades in Italy. On the one hand, labor market conditions for women have sensibly improved from the sixties to the early-twenties (Mussida and Picchio 2014). On the other hand, beginning in the nineties, several pension reforms have been adopted in an attempt to keep pension expenditures under control. Most importantly, reforms in Italy have mostly reduced the generosity of the old pension system through the transition from a Defined-Benefit (DB) and *redistributive* pension plan to a (notional) Defined-Contribution (DC) and *actuarially fair* scheme.^1^ As far as women are penalized, on average, due to both career discontinuities and lower earnings, the transition from a redistributive to an actuarially fair scheme may clearly reduce, or even offset, the effect of bettering labor market conditions for women.

In this paper, we investigate the dynamics of the gender gap at retirement^2^—by cohort and by year of retirement—in Italy, with particular emphasis on its determinants. Cohorts from 1936 to 1965—retiring from 1980 to 2027—are considered to investigate the impact of the transition from the DB to the DC scheme. The time span of the analysis has been defined in such a way as to capture both driving forces which may have influenced the dynamics of gender inequality at retirement in recent times. We use SHARELIFE data (wave 3 and wave 7) on the real working life of individuals aged 50 years old at least in 2008 (last chronological year in wave 3) and in 2016 (last chronological year in wave 7). To our knowledge, this is the first attempt to estimate the dynamics of the gender gap at retirement in Italy by using real data.

Our analysis is mostly, but not entirely, retrospective. We consider both the population of retirees from 1980 to 2008 in wave 3, and from 1980 to 2016 in wave 7, as well as the population of still-in-job individuals in 2008 (wave 3) and 2016 (wave 7) who are expected to meet (statutory or blended) eligibility requirements for an old-age or a seniority pension within 2027. As for the prospective analysis, life expectancy coefficients for workers retiring in the year 2021 onward are obtained by projecting observed mortality rates, according to the Lee–Carter model (Lee and Carter 1992), with data provided by the Human Mortality Database (Berkeley Human Mortality Database 2020).

For our purposes, we adopt a counterfactual analysis by which the *actual* distribution of (yearly) pension benefits—for each cohort and year of retirement—is compared to the corresponding *virtual* distribution obtained under the hypothesis of a fully DC and actuarially fair scheme. Remarkably, in order to emphasize the effect of the changing pension scheme, we focus on the *insurance mechanism* characterizing pension systems, i.e., the sole population of individuals satisfying eligibility requirements for an old-age or seniority pension (or blended criteria). Differently, first-tier pensions (e.g., basic, minimum and means-tested old-age social assistance payment) are excluded from our analysis, since these belongs to the sphere of *social assistance*, whose objectives and rules go well beyond the definition of a pension annuity from a contribution career.^3^

We find a U-shaped dynamics for the Gender Gap in Pension (GGP) across cohorts, where the first and descendant part of the U-shape is characterized by both (i) improving labor market conditions for women and (ii) rich-to-poor redistribution due to the overwhelming application of the DB scheme. In the subsequent and ascendant part, we find that, even if the absolute gender gap in terms of mean pension benefits is almost unchanged, the same gap is increasing in relative terms (i.e., percentage points) because of the reduction of mean pension benefits originating from the progressive application of the less generous DC scheme.

Unexpectedly, for workers retiring after 2016 with an old-age or seniority public pension, we find that the gender gap at retirement is slightly higher than what it would have been if a perfect actuarially fair DC scheme were applied. Basically, since the DB and the DC scheme apply ‘pro-rata’ during the transition to a fully DC system, women are found to be additionally penalized (relative to males) by (i) a higher starting working age, and (ii) career discontinuities in the early working life, which reduce for women the number of years of contribution under the more generous DB scheme (more than for men in the same cohort retiring in the same year).

Even if the GGP is a standard metric for the measurement of the gender gap at retirement, we also apply the decomposition of Generalized Entropy (GE) inequality indexes in terms of between-group (or between-gender) and within-group (or within-gender) inequality. Indeed, this approach provides additional information on the dynamics of within-gender and overall inequality at retirement, which allows for a better understanding of the determinants of the observed dynamics of the gender gap at retirement. Most importantly, it allows for measurement of how the redistributive impact of the DB scheme, independently from its indirect effect on gender disparities, has changed across cohorts and years of retirement.

The results of the GE decomposition confirm both the U-shaped dynamics of the (relative) gender gap at retirement, as well as the penalization of women in the pro-rata mechanism due to higher starting working age and career discontinuities in early working life. In addition, the GE decomposition reveals that the share of overall inequalities originating from gender disparities at retirement remains mostly unchanged after 2016, thereby arresting the decreasing trend observed in the previous decades.

The paper is organized as follows. In Sect. 2, we discuss major pension reforms that occurred in Italy from the nineties, with a special emphasis on the transition from the DB to the DC pension scheme. In addition, existing evidence on the dynamics of the gender gap at retirement are reported for Italy and other major OECD countries. In Sect. 3, we discuss the main characteristics of the SHARE database, as well as the methodology and the results of our analysis. Section 4 provides concluding remarks.

## Gender Gap at Retirement in the Italian Pension System

In this section, we first discuss the main characteristics of the Italian pension system, with particular focus on the reform process that has determined the ongoing transition from the old DB to the new Notional Defined Contribution (NDC) scheme.^4^

In the second part, we discuss the existing literature on the gender gap at retirement, which is based on both official reports and research papers. For our purposes, special emphasis is placed on recent papers discussing the recent and the expected evolution of the gender gap at retirement in Italy.

### Pension Reforms in Italy: From DB to NDC System

The Italian pension system is based on a pay-as-you-go (PAYGO) mechanism, where pension benefits of retirees are financed by pension savings (hereafter contributions) paid by the working age population. During the nineties, the Italian pension system underwent a major reform process, moving from a DB to a NDC system, while maintaining the PAYGO financing mechanism.^5^

Up until 1992, according to the DB formula, pension benefits were determined by *pensionable earnings*, defined as the average wage earned in the last years before retirement,^6^ by the number of *years of contribution* at retirement (topped at 40 years), and by the *accrual rate* of 2% for each year of contribution. Workers became eligible for retirement if they paid either at least 35 years of contribution (known as seniority pension), or they were 55/60 years old (female/male) with at least 15 years of paid contributions (known as old-age pension). In addition, an indexation mechanism to price inflation and real wages was implemented for the adjustment of the first pension benefit in later life, after retirement.

Most importantly, the DB scheme was characterized by several redistributive tools like floors, ceilings, and redistributive accrual rates. These have been slightly amended over time beginning in the eighties. *Floors* were established to raise pension benefits to a minimum amount, if they were lower. On the other hand, *ceilings* were used to identify an upper bound for pensionable earnings to be accounted for in the calculation of the first pension. The accrual rate was—and still is—*redistributive*; it was set at 2% for pensionable earnings below a reference level legislated annually (inflation-adjusted), at 1.50% for pensionable earnings between the reference level and 1.33 times the reference level, at 1.25% between 1.33 and 1.66 times the reference level, and at 1% above 1.66 times the reference level (Brugiavini and Peracchi 2003).^7^

In 1992, to account for inter-generational inequity originating from the financial burden for the youngest generations caused by the excessive generosity of the pre-1992 system, the Amato Reform (Law 503/1992) sensibly modified the DB scheme by setting different and more stringent eligibility rules and by gradually increasing the number of years over which pensionable earnings were to be computed.^8^ Specifically, while preserving the old DB scheme for years of contribution until 1992 (so-called “Quota A”), a modified DB scheme is introduced for years of contribution after 1992 (so-called “Quota B”). As for the latter, a distinction is made between workers with more and those with less than 15 years of contribution in 1992. For workers with at least 15 years of contribution, pensionable earnings are defined up to the average wage in the last ten years (15 for self-employed workers). Instead, for those with less than 15 years of contribution in 1992, pensionable earnings are computed as the average wage in the last five years (10 for self-employed workers). In addition, the set of accrual rates—still redistributive in its nature—is slightly revisited for contributions after 1992 (i.e. “Quota B”), mostly through the introduction of a new earning bracket for very high pensionable earnings.^9^

In 1995, the Dini reform (Law 335/1995) modified the structure of the Italian pension scheme by marking a gradual transition from a DB to a DC benefit formula. In order to allow for a progressive implementation of the DC scheme across cohorts, different eligibility rules and benefit formulas are established depending on the years of contribution achieved by each worker in 1995. Specifically, a separating line is introduced among elder, middle-aged and early workers, which is currently still in force.

For workers entering the labor market after 1995 (early workers), the sole NDC benefit formula applies by which the first pension benefit is calculated from the total amount of contributions paid during the entire working life—notionally capitalized at the GDP nominal growth rate—and converted into an actuarially fair annuity through the application of an age-increasing Transformation Coefficient^10^ (Daminato and Padula 2020). Specifically, the conversion into a pension annuity is determined by the Expected Pension Period Duration (EPPD), i.e., by the expected number of years for which pension benefits must be paid (Coppola et al. 2020), provided that the life expectancy of both retirees and survivors is accounted for (see Sect. A.4 in the appendix for major details). According to the current legislation, Transformation Coefficients must be updated every two years depending on demographic tables and long-term trend of GDP officially provided by the Italian National Institute of Statistics (ISTAT).

For workers with at least 18 years of contribution in 1995 (elder workers), the defined benefit formula applies, but pensionable earnings after 1992 are computed averaging over a longer period according to the modified DB formula set up by the Amato reform.^11^

Finally, for workers with less than 18 years of contribution in 1995 (middle-aged workers), pension benefits are computed according to a pro-rata mechanism (hereafter Dini pro-rata) by which the first part of benefit referring to the pre-1995 years of contribution is computed according to the modified DB formula, while the NDC scheme applies for contributions paid afterwards.^12^

In 2011, the Monti-Fornero Reform (Decree-Law 201/2011) has adopted several measures—fully applicable to labor market entrants from 1996 onward—promoting more stringent but more flexible eligibility requirements, as well as more inter-generational equity. As for the standard old-age pension, provided that (i) contributions have been paid for at least 20 years and (ii) the pension benefit is no less than 1.5 times the old-age social assistance payment, under the new system the age of retirement has been gradually increased for men and women, until 2021, when no categories of workers are able to retire before the age of 67. Similarly, the years of contribution needed to be eligible for a seniority pension (early retirement) has increased gradually for men and women, until 2021, when eligibility was fixed at 41/42 (women/men) years and 10 months.^13^ Moreover, in line with previous legislative actions, the Monti-Fornero reform established that both age and seniority requirements must be periodically adjusted according to life expectancy coefficients published by ISTAT.

As for inter-generational equity, the Monti-Fornero reform has also amended the modified DB scheme established by the Dini reform for workers with more than 18 years of contributions on 31 December 1995 (elder workers). To reduce the excessive generosity of the old DB pension scheme for these workers, the application of the NDC has been introduced starting with contributions accrued from 1 January 2012 onward. As such, for elder workers still in the labor force on 31 December 2011, a pro-rata mechanism (hereafter Fornero pro-rata) applies as well, which is not to be confused with the Dini pro-rata.

Finally, for our purposes it is worth mentioning the Decree-Law 4/2019 and the Budget Law for 2022, by which more favourable early retirement rules (i.e., “Quota 100” and “Quota 102”) have been exceptionally introduced for workers retiring, respectively, until 2021 and in 2022. As for “Quota 100”, workers are entitled to retire once the 100-year threshold is achieved by summing 62 (or more) years of age and at least 38 years of contribution; the age threshold is set to 64 for “Quota 102”.

### Gender Gap at Retirement: Existing Evidence

In the existing literature, the gender gap at retirement is usually measured in terms of GGP, which is defined as the percentage by which women’s average pension is lower than men’s. It is computed as one minus women’s average pension income divided by men’s average pension income.

In 2017, according to Dessimirova and Bustamante (2019), the average GGP in the EU-28 was 35.7% for the population of retirees aged 65–79 years old, with a 35.8% GGP in Italy.^14^ As far as possible, it is interesting to compare this evidence with previous findings for the population of retirees aged more that 65 years old in Bettio et al. (2013). According to the latter, the GGP in the EU-27 was 39.1% in Europe, but 31.0% in Italy, casting some concerns about the possibility of opposite dynamics of the GGP in Italy with respect to the rest of Europe.^15^

The various factors determining the GGP can be grouped into two categories: (i) the design of the pension system and (ii) the working career of individuals.

In regards to the first category, pension systems are usually considered as gender neutral as most of the rules and provisions are the same for men and women. However, it has been observed that, since the reforms in the 1990s, pension systems have shifted towards contribution-based occupational schemes which are likely to increase the GGP due to greater penalization for women (Chlon-Dominczak 2017, Samek Lodovici et al. 2016).

With regard to the second aspect, i.e., the working career of individuals, there are different elements characterizing individuals’ working life. According to official Eurostat statistics (Leythienne and Pérez-Julián 2021), the average Gender Pay Gap (GPG) in the EU-27—i.e., the difference in average gross hourly wage between men and women across the economy—in 2018 was estimated to be 14.1%, slightly lower than in 2015 (15.5%). From the same report, the gap for Italy was found to be 4.7%, showing a decreasing pattern with respect to the 7.9% observed in 2014. The same metric was estimated to be 18% in 2019 for the U.S. (Barroso and Brown 2020).

Notably, a lower GPG does not necessarily indicate greater gender equality. Rather, a lower gap can be a consequence of lower labor market participation of women (e.g., in Italy the employment rate of women in 2018 was 53.1% compared with 72.9% for men). According to the European Institute for Gender Equality (EIGE 2018), women spend more years in unpaid employment than men because of their greater role in the care of children or elderly parents. This report highlights that 15.0% of 15-to-64 years old inactive women are inactive for reasons related to providing care. Likewise, the average employment rate for mothers aged 20–49 with a young child (younger than 6 years old) is 65.4% in comparison to 91.5% of fathers. Women also differ from men for work intensity; they work part-time or in fixed-term employment more often than men. Within the EU, data shows that 31.3% of women work part-time in comparison to 8.7% of men. Furthermore, on average, 12.7% of women work in temporary contracts compared to 10.7% of men (EIGE 2018). All-in-all, women are found to be penalized in terms of both discontinuous careers and average earnings.

As for Italy, due to the increasing concern for the ongoing transition to the NDC scheme, several studies have explored how gender roles, which are changing over time, are expected to interact with the shifts in pension policies.

Zanier and Crespi (2015) provide an overview of the phenomenon of increasing gender inequalities that happen at old age regarding women’s pension. Their paper is one of the first critical reviews of the effect of gender inequalities in relation to pension gaps in Italy. Throughout a European overview of pension gender gap, focusing in particular on Italy, the authors analyze the reasons behind gender-biased pension levels and how their cumulative effects result in significant gender gaps. Specifically, they conclude that welfare and social policies in Italy do not account for the needs of the family and of women, which results in being unable to provide necessary answers to the growing concern for gender gap.

Leombruni and Mosca (2012) provide the first results on how the GPG evolves during the entire working career of individuals in Italy and how it translates into a further gap in terms of lifetime income. They exploit an original database—obtained from two different administrative archives, the Work Histories Italian Panel (WHIP) and the National Social Security Administration (INPS) Contribution Accounts (CA)—to micro-simulate the entire working career of a sample of people who retired in 2004. Hence, the authors document how the pay gap constantly widens with age and how women tend to cumulate a lower number of eligible working years. They find that both these gaps have an impact on the pension calculation, so that gender differences become even higher at retirement. They show that the pension system partially counterbalances labor market effects, implying lower differences in lifetime incomes. Nevertheless, due to the current transition to an actuarially neutral system, this effect tends to disappear. This, in turn, poses some concerns about the prospects of the gender lifetime income gap.

Lorenti et al. (2019), by estimating the Working Life Expectancy (WLE) (i.e., the expected years of life spent in employment) find a large and increasing heterogeneity in the length of the working life. They analyze trends in WLE in Italy and show that during the recent financial crisis, the gender gap in WLE increased, with a difference of 3.5 years between men and women in 2012–2013. They find that discontinuous careers and the limited accumulation of contributions may result in inadequate pension benefits later in life. From a policy perspective, they conclude that it is crucial for Italy to increase employment levels at all ages, particularly among women, young people, and people who live in the South.

## Analysis

### Data

This paper uses data from SHARE (The Survey of Health, Aging and Retirement in Europe) Wave 3 (10.6103/SHARE.w3.800) and Wave 7 (10.6103/SHARE.w7.711), see Börsch-Supan et al. (2013) for methodological details.^16^ The collection of data for these waves started, respectively, in 2009 and 2017 reporting information updated to chronological years 2008 and 2016. Only the population aged 50 years and greater were interviewed.^17^ We use both wave 3 and wave 7—including different respondents^18^—in order to achieve a sufficient number of observations for the purposes of our analysis.^19^

As it stands, wave 3 and wave 7 are the only two waves with the SHARELIFE questionnaire, which focuses on people’s life histories including all of the important areas of respondents’ lives, ranging from partners and children to housing and work history with detailed questions on health and health care. Most importantly, for each job position lasting at least six months, the set of retrospective employment questions includes data on employment spells (starting/ending year of each job position), employment status, job characteristics, income, retirement benefits, and employment after retirement. Data are also collected about the typology of contribution plans (public, occupational, private, individual), as well as about the type of public pension benefits (old-age or seniority-based, sickness, disability, survivor, social assistance, etc.). At present, this is the most important dataset for the reconstruction from real data of the contribution career of retired, or almost retired, workers in the EU (Alessie et al. 2013).^20^

Wave 3 and wave 7 consist of 2,528 and 4,571 observations for Italy, respectively. For our purposes, we only consider respondents who paid, or pay if still in job, contributions to a public pension plan, and receive old-age or seniority benefits if already retired. In addition, the sole population of workers achieving the minimum years of contribution for pension entitlement (20 years) are considered. The selection of the sole respondents receiving an old-age or seniority benefit from contributions to a public pension plan allows to focus on the sole impact of the *insurance* scheme (DB and/or DC) independently from *social assistance* at retirement usually pursued through first-tier benefits like survival pensions, minimum retirement benefits, etc.

We also restrict our sample to individuals born after 1935, since these workers are immediately involved in the transition from the fully DB to the NDC pension scheme. Still-in-job respondents are included if expected to meet minimal eligibility requirements for retirement no later than 2027 (old-age pension, or seniority pension, or ‘Quota 100’ from 2019 to 2021 or ‘Quota 102’ in 2022). For these individuals, salaries in 2008 (last chronological year in wave 3) and in 2016 (last chronological year in wave 7) are assumed to grow at the earnings growth rate reported by the last available official statistics.^21^ To avoid strong assumptions on the future dynamics of earnings, we limit the simulated part of the analysis (prospective analysis) to 2027; hence, for two decades (2009–2027) in wave 3 and for one decade (2017–2027) in wave 7, job positions are assumed to preserve the same characteristics held at the time of the interview (resp. 2008 and 2016), and the pension system is assumed to retain the same eligibility requirements as in 2021 (except for ‘Quota 100’ ending in 2021 and ‘Quota 102’ in 2022).

After restricting the data set (i) to respondents joining a public pension plan belonging to the 1936 cohort onward, (ii) to retirees and still-in-job respondents expected to meet pension eligibility requirements no later than 2027, and (iii) after dropping observations reporting missing, or unknown, or refusal values (for at least one among starting/ending period of the employment spell, salary in the employment spell, wage currency, and gender), the final data set consists of 457 observations from wave 3 (298 males and 159 females) and 847 observations from wave 7 (545 males and 302 females), summing up to 1304 observations.

Due to the limited number of observations, we define groups of individuals through a disjoint and exhaustive partition of the population by (i) year of birth and (ii) year of retirement. More specifically, we consider three consecutive intervals for the year of birth (cohort): 1936–1945, 1946–1955, and 1956–1965. Additionally, we consider three consecutive intervals for the year of retirement: 1980–2005, 2006–2016, and 2017–2027. As a result, we obtain 9 cells, where the sole cells on the main diagonal of Table 1 are characterized by more than 50 observations for both males and females (for the same cohort/retirement partition by wave, see Table 8, Appendix A.1).

**Table 1:**
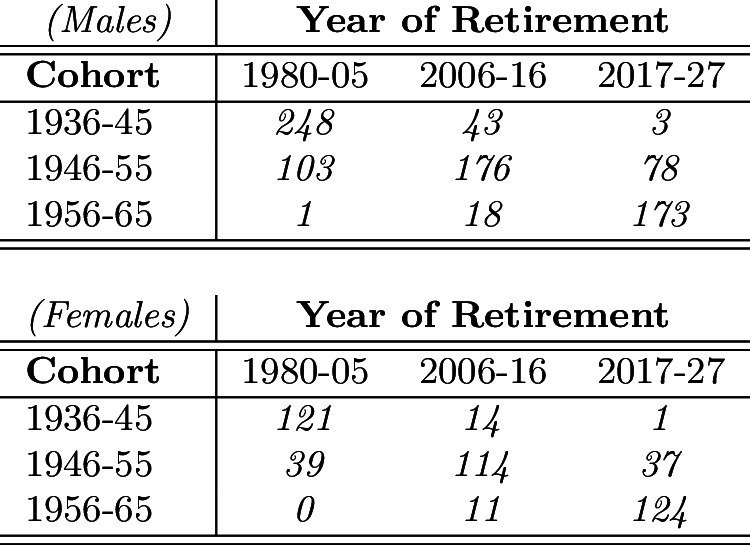
Number of observations by cohort, year of retirement and gender

For this reason, we confine our analysis to the three cells on the main diagonal, namely Cell 1 = (1936–1945; 1980–2005), Cell 2 = (1946–1955; 2006–2016), and Cell 3 = (1956–1965; 2017–2027). This excludes from the analysis most of the cases of very early and very late retirement. Also, notice that Cell 3 is composed by still-in-job respondents from both wave 3 and wave 7, whereas a few still-in-job respondents from wave 3 may populate Cell 2.

Main descriptive statistics for Cell 1, Cell 2, and Cell 3 are reported in Table 2 (see Table 9, Appendix A.2, for descriptive statistics by wave). On average, the population in each of the three cells consists of males and females born in 1940–1941, 1950, 1959, respectively, whose working life started on average in the early sixties, seventies and eighties. On average, males enter the labor market younger than females, with a decreasing gap over time.^22^

In Table 2, we also indicate the net monthly income from main job (identified as “main” by the respondent) and the average years of contribution by gender. This information allows us to calculate both the GPG for the sole main job, i.e., the ratio between the absolute gender gap in terms of average wages and the average wage of males, as well as the Gender Gap in Seniority (GGS), i.e., the ratio between the average absolute seniority gap at retirement and the average seniority of males.^23^

**Table 2:**
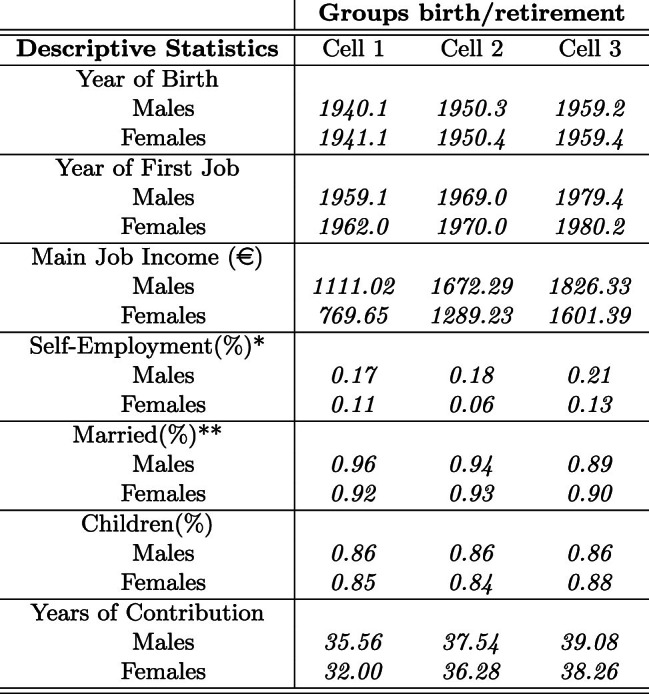
Descriptive statistics by gender

$^*$ If self-employed for one employment spell at least

$$^{**}$$ If married at least once

**Table 3:**
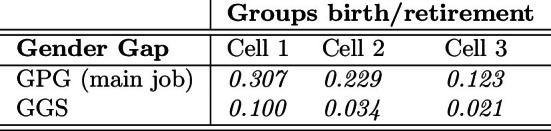
Gender pay gap (main job) and gender gap in seniority

As reported in Table 3, the GPG has sensibly reduced across cells, at least for the main job. In addition, the GPG is found to be even lower for the sole population of still-in-job respondents in 2008 (wave 3) and in 2016 (wave 7), respectively, $GPG=0.044$ and $GPG=0.039$. This is consistent with existing evidence for Italy discussed in Sect. 2.2. From Table 3, we also observe the dynamics of the GGS, which confirms the relevant increase in women’s participation in the labor market from Cell 1 to Cell 2 (see Table 10, Appendix A.3, for the same gaps by wave).

Notice that, for each respondent and employment spell, labor earnings are reported either as wages-in-job (for employees), or as incomes (for self-employment), by indicating the amount of earnings received at the beginning of each job (spell). As far as job positions lasted less than six months are omitted in SHARE, the amount of accrued savings might be undervalued for some workers, especially for gig workers. Remarkably, respondents are required to indicate the net value of their starting earnings, implying that, as discussed in the next section, gross incomes must be simulated from net incomes in order to reconstruct the contributions paid by workers during their entire working life.

### Methodology

Whereas the design of the Italian DB scheme is redistributive while the NDC is not, and provided that females accrue less contributions than males on average (due to lower labor earnings and career discontinuities), one may reasonably expect the transition from the DB to the NDC scheme to exacerbate *ceteris paribus* the GGP. On the other hand, the observed reduction of the GPG, especially during the sixties and the seventies, may push the GGP in the opposite direction, to offset the previous effect.

To capture both the dynamics of the GGP and the impact of the progressive replacement of the DB with the NDC scheme, we implement a counterfactual analysis by which the *actual* distribution of pension benefits reported by respondents is compared to the *virtual* distribution obtained by applying the sole NDC benefit formula to the entire working life of individuals in our population (Cells 1–2–3).

Let the GGP in the ith Cell be defined as

1 $$\begin{aligned} GGP_i=1-\frac{\mu _{i}(F)}{\mu _{i}(M)} \end{aligned}$$

with $\mu _i(F)$ and $\mu _i(M)$ indicating the average pension benefit in the ith Cell of females and males respectively.

The dynamics of the *actual GGP* from Cell 1 to Cell 2 and from Cell 2 to Cell 3 reveals the overall effect determined altogether by the evolution of women’s participation in the labor market and by the progressive replacement of the DB with the NDC scheme. Differently, as far as *virtual GGPs* across cells are computed by holding fixed the pension scheme, the dynamics of the virtual GGP captures the sole impact of the evolution of women’s participation in the labor market during the last decades.

Taken together, the difference between the actual and the virtual GGP measures the impact on the real population of the redistributive trait of the DB scheme in the absence of behavioral responses, i.e., holding fixed the working life of individuals. Reasonably, to the extent that the DB scheme is progressively replaced by the NDC scheme over time, the redistributive effect of the DB scheme is expected to be greater in Cell 1, and then to decrease across cells (from Cell 1 to Cell 3).^24^

The GGP-based counterfactual analysis discussed above allows us to highlight the contribution of different driving forces to the observed trend of inequality (gap) between gender. However, for a more complete understanding of this trend, one may want to also consider the impact of these driving forces on the inequality within gender, i.e., among males or females only, as well as on overall inequality among the population of retirees by cohort and by year of retirement. To account for the dynamics of within-gender inequalities, we calculate Generalized Entropy (GE) measures of inequality, which are known to be additively decomposable in terms of within-group (within-gender) and between-group (between-gender) inequality (Shorrocks 1980).

Let $b_{ij}$ be the pension benefit (actual or virtual) of the jth retiree in the ith Cell, the class of GE inequality measures is defined as

2 $$\begin{aligned} GE_i(\alpha )=\left\{ \begin{array}{ll} \frac{1}{n_i\alpha (\alpha -1)}\sum ^{n_i}_{j=1}\left( \left( \frac{b_{ij}}{\mu (b_i)}\right) ^\alpha -1\right) &{}\quad \alpha \ne 0,\\[4pt] \frac{1}{n_i}\sum ^{n_i}_{j=1}\frac{b_{ij}}{\mu (b_i)}\ln \frac{b_{ij}}{\mu (b_i)}&{}\quad \alpha = 1,\\[4pt] -\frac{1}{n_i}\sum ^{n_i}_{j=1}\ln \frac{b_{ij}}{\mu (b_i)}&{}\quad \alpha =0 \end{array} \right. \end{aligned}$$

The parameter $\alpha$ regulates the weight given to distances between pension benefits at different parts of the income distribution; the greater is $\alpha$ the more the index is sensitive to pension benefits at the top of the distribution (e.g., GE increases more when a higher pension benefit increases). Vice versa, the lower is $\alpha$ the more the index is sensitive to pension benefits at the bottom of the distribution.

Several well-known inequality metrics can be obtained from the class of GE measures by letting $\alpha$ change: e.g. *GE*(0) is known to be the mean log deviation index; *GE*(1) corresponds to the Theil index; *GE*(2) is half the square coefficient of variation. Most importantly, each of the GE measures is additively decomposable, in that each of these indices can be reformulated in terms of within-group and between-group inequality as follows

3 $$\begin{aligned} GE_i(\alpha )= & {} GE^W_\alpha +GE^B_\alpha \nonumber \\= & {} \sum ^2_{k=1} \frac{n_{ik}\mu _{ik}}{n_i\mu _i}\left( \frac{\mu _{ik}}{\mu _i}\right) ^\alpha GE^k_i(\alpha ) + \frac{1}{\alpha (\alpha +1)}\sum ^2_{k=1}\frac{n_{ik}\mu _{ik}}{n_i\mu _i}\left( \left( \frac{\mu _{ik}}{\mu _i}\right) ^\alpha -1\right) \nonumber \\ \end{aligned}$$

with $k=(F,M)$ for female and males respectively, and $GE^k_i(\alpha )$ indicating inequality in group *k* of the ith Cell.

As compared to the GGP, the group-decomposition of inequality measures provides information on both the between-gender gap (between-group inequality) and the within-gender gap (within-group inequality), as well as on the overall inequality. In a dynamic perspective, additional information on the evolution of within-group and overall inequality may allow for a better identification of the contribution of generic (non gender-specific) rich-to-poor redistribution, independently from its impact on the dynamics of the gender gap. Intuitively, concordant variations of the within-group and between-group inequality components are naturally expected in the case of generic rich-to-poor redistribution, whereas this is not necessarily the case for gender-specific redistribution.^25^

Also, it is worth observing that the dynamics of the two metrics (GGP and GE) may differ in size depending on what average pension benefit—the one of females or males—is changing more, since the GGP is more sensitive to variations of the average pension benefit of males. This is because the GGP is obtained by dividing the absolute (monetary) gender gap by the average pension benefit of males, whereas both males and females’ average pension benefits are equally considered in GE measures.

#### Pension Savings at Retirement: Main Assumptions

The counterfactual analysis we propose is based on the reconstruction of the working career of ‘real’ individuals from their ‘real’ working life. This allows us to obtain a more realistic picture of the current and expected evolution of the gender gap at retirement in the near future. However, as much as the reconstruction of the entire working career is a very information-demanding process, simplifications and generalizations are inevitably required. Some of them are almost natural and standard practice in the field of pension systems; some others originate from limited information on the working history of respondents and are more relevant. In what follows, we discuss the details of the assumptions adopted in our analysis.

First, in the SHARELIFE database, labor earnings for each employment spell are reported for the first year only. This is particularly insidious for long-lasting job positions. To account for earnings’ progression in the same job, the first income in each spell is capitalized at the earnings growth rate—differentiated by macro sector—from official statistics of the Italian National Institute of Statistics (ISTAT).^26^ By merging the work-type classification in SHARELIFE (wave 3 and wave 7) and in ISTAT’s official statistics, we can disentangle the following macro sectors: agriculture, hunting, forestry, fishing; primary and secondary sector; transport services; public administration; wholesale and retail trade; general economy. Missing data from 1950 to 1955 are simulated by implementing a 5 yearly moving average process. For years after 2015, as discussed later in this section, we refer to periodical data releases from ISTAT.

Second, the SHARELIFE database reports the net value of labor earnings whereas, according to the Italian legislation, mandatory contributions are calculated with respect to gross labor earnings, so including income taxes. To calculate income taxes, we assume that the tax base consists exclusively of incomes from wages, self-employment, and small firms profits.^27^ The Italian personal income tax, namely IRPEF, underwent many reforms from 1950 onward (e.g., 14 acts since the ‘big’ tax reform adopted with Presidential Decree 600/1973).^28^ As such, in our analysis we opted for a partial reconstruction of the evolution of the Italian tax system by identifying three different time periods. The time span of these periods has been determined in such a way as to keep as much homogeneity as possible. For labor earnings from 1951 to 1992, income taxes are calculated by considering tax brackets and marginal tax rates in the 1974 tax system. For the time interval 1992–1997, we apply tax brackets and marginal tax rates in force in 1992, as modified by the Amato reform. Finally, for taxes from 1998 to 2027, we apply the 2016 tax system in force from 2007.^29^ Due to the lack of information in SHARE, tax expenditures are neglected except for the tax deduction of 1/3 of total amount of pension savings,^30^ whose rate is fixed at the 33% and 20%^31^ for employees and self-employed workers respectively.

Third, our analysis is mainly but not entirely retrospective in that we also consider still-in-job respondents in 2008 (wave 3) and in 2016 (wave 7), who are supposed to retire no later than 2027 (prospective analysis). While the actual pension benefit is directly reported by retirees in the SHARE database, for still-in-job respondents we calculate the actual pension benefit—to be received after 2008 (wave 3) and 2016 (wave 7) up to 2027—assuming that (i) individuals choose to retire at the first chance according to eligibility requirements in force each year from 2009 onward (holding fixed the 2022 system up to 2027),^32^ (ii) job positions in 2008 (wave 3) and 2016 (wave 7) are preserved up to retirement,^33^ (iii) earnings increase, by macro sector, at the observed earnings growth rate until 2020 and at the average 2016–2020 earnings growth rate in case of retirement after 2020.^34^

Fourth, the official Transformation Coefficients used for the calculation of pension benefits in the NDC scheme (formally updated by the government in charge every two years) are currently available up to 2022. Hence, to calculate actual and virtual pension benefits for individuals retiring after 2022 (prospective analysis), we also need to update the Transformation Coefficients in such a way as to account for the dynamics of life expectancy. In the actuarial literature, a variety of alternative methodologies for projecting mortality have been proposed (Lee and Carter 1992, Booth and Tickle 2008, Russolillo et al. 2011). Following the official estimation strategy implemented by ISTAT, we refer to the Lee–Carter model and apply it to mortality data provided by the Human Mortality Database (HMD). The study is performed on the Italian male and female populations ranging from 1960 to 2017, for ages from 0 up to 102 years, considered by single calendar year and by single year of age (see Sect. A.4 in the appendix for details on the estimation strategy). Hence, survival and death probabilities are derived from 2018 up to 2030 and, in turn, Transformation Coefficients for biennium’s 2023–2024, 2025–2026, and 2027–2028 are obtained by using as baseline years 2022, 2024 and 2026, respectively.^35^

Finally, given labor market earnings of individuals during their entire working life, the application of the NDC scheme to calculate the virtual distribution of pension benefits (under the hypothesis of a fully NDC scheme in each cell) poses an additional threat for the capitalization of contributions paid from 1950 to 1962 and for 2020 onward. Indeed, ISTAT provides official capitalization rates to be applied in the NDC scheme exclusively for years from 1962 to 2021, whereas we also need the capitalization for the calculation of virtual pension benefits of individuals who entered the labor market before 1962 and for those individuals (still-in-job) expected to retire after 2020. To fill this gap for both time intervals, we simulate the same procedure implemented by the ISTAT by taking the 5 yearly moving average of the GDP growth rate as reported in the official statistics.^36^

### Results

After reconstructing the entire working career of individuals by cohort and year of retirement, we calculate (i) the actual pension benefit at the first year of retirement for still-in-job respondents (which is instead reported for retirees), and (ii) the virtual pension benefit for each respondent. This computation involves the application of different benefit formulas, either DB or NDC, along the working career. To account for possible outliers, observations below and above the 1st and the 99th centile in the actual and virtual pension distributions have been dropped (e.g., Jarvis and Jenkins 1998). As a result, the sample size consists of 369 units in Cell 1, 290 units in Cell 2, and 276 units in Cell 3.

Following the Dini and the Monti-Fornero reforms discussed in Sect. 2.1, the NDC scheme progressively replaces the DB in the application of the benefit formula, even if our subsample does not include any individual retiring with a fully NDC scheme. As reported in Table 4, in Cell 1, 364 out of 369 retirees (98.6%) obtain a fully DB pension. Instead, in Cell 2, 161 out of 290 retirees (55.5%) get a fully DB pension due to more than 18 years of contribution in 1995 and retirement before 2012; for 111 out of 290 retirees (38.3%) the NDC applies from 2012 up to retirement; for 18 out of 290 retirees (6.2%) the NDC applies from 1996 up to retirement. Finally in Cell 3, for 79 out of 276 retirees (28.6%) the NDC only applies after 2012 to retirement, whereas for 197 out of 276 (71.4%) the NDC applies from 1996 up to retirement.

**Table 4:**
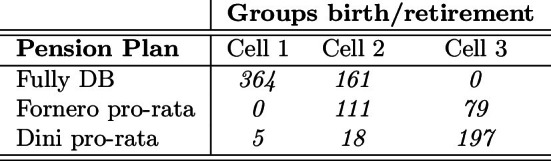
Number of observations by pension plan

As we said above, the dynamics of the composition of the population in each cell with respect to the pension scheme in force is key for a proper understanding of main results discussed in this section. In addition, as for the composition of cells, it is worth recalling that the last part of the working life is simulated for the population of still-in-job individuals; this happens to be the case for 49 observations from wave 3 falling in Cell 2 (16.9%) and for all the 276 observations in Cell 3 (18 and 258 from wave 3 and wave 7 respectively).

For each cell, actual and virtual (gross) mean pension benefits are reported in Table 5.^37^

From the first panel in Table 5 (Cell 1), the gross actual pension benefit is found to be higher than the virtual one for both males and females, with males receiving a greater benefit than females. This is just what one may expect since the NDC scheme is known to be less generous than the Italian DB pension formula, and females usually accrue less contributions than males during their working life. Not surprisingly, the same evidence is confirmed for Cell 2 and Cell 3, even if the gap between actual and virtual benefits is found to be sensibly lower in Cell 3, for both males and females, due to the larger application of the NDC scheme to the working career of these individuals.

**Table 5:**
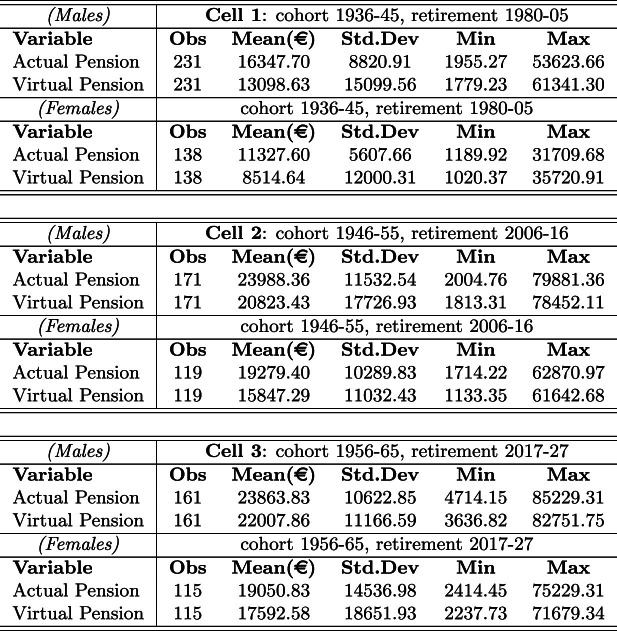
Mean yearly (gross) pension benefits by gender for Cells 1–2–3

As concerns the dynamics of actual pension benefits across cells, it is worth observing that, for both males and females, actual pension benefits are first increasing from Cell 1 to Cell 2, then slightly decreasing from Cell 2 to Cell 3. This is mostly the result of two driving forces moving in the opposite direction. On the one hand, labor market earnings have sensibly increased from the sixties, with a remarkable increment due to the transition from the Italian Lira to the Euro. On the other hand, the progressive replacement of the old DB scheme with the less generous NDC scheme tends to reduce the mean pension benefit. While the first effect is overwhelming in the transition from Cell 1 to Cell 2, the latter effect becomes dominant from Cell 2 to Cell 3.

As for the dynamics of the virtual pension benefit, this is sensibly increasing across cells due to the sole impact of increasing wage, especially for cohorts starting to work in the early sixties (see Table 3). Most importantly, the positive variation occurs at a higher rate for females since improving labor market conditions for women has permitted greater accrual of contributions.

#### GGP Analysis

From Table 5 and Eq. (1), we obtain the actual and virtual GGPs reported in Table 6.

**Table 6:**
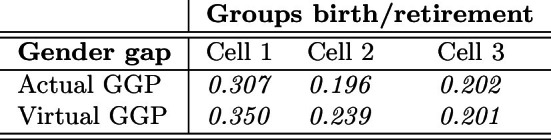
Gender gap in pension (GGP)

From the comparison between the actual and the virtual GGP in Cell 1, the redistributive impact of the DB pension scheme, compared to the actuarially fair NDC scheme, is clearly highlighted. Indeed, as we mentioned above, the population of retirees in Cell 1 is almost entirely characterized by the application of a fully DB scheme, so the redistributive impact is almost entirely captured through the comparison to the virtual GGP (fully NDC) in Cell 1. We also observe a very high actual GGP in Cell 1, which is originating from both high wage differentials and career discontinuities of females (see Table 3).

In Cell 2, as reported at the beginning of this section, the population is mostly characterized by a minor application of the NDC. This mostly applies to contributions paid after 2011 by some retirees. Here, the actual GGP is found remarkably lower than in the previous cell due to improving labor market conditions for females. This is clearly observable both from the reduction of the GPG in main job and from the conspicuous reduction of the GGS (Table 3), which decreases from 10.0% to 3.4% from Cell 1 to Cell 2. Consistently, the virtual GGP sensibly falls from 0.350 to 0.239. As for the redistribution induced by the pension scheme, in Cell 2 the actual GGP is found to be, once again, lower than the virtual GGP, in that the application of the DB scheme is still overwhelming in this cell.

The results of the GGP analysis are different when considering Cell 3, for two reasons at least. First, the actual GGP is unexpectedly increasing from Cell 2 to Cell 3. This result is even more surprising if one considers that both the GPG and the GGS are both decreasing from Cell 2 to Cell 3 (Table 3). This unexpected finding is motivated by both (i) the transition to a less generous pension plan (the NDC scheme) which causes a reduction of the denominator (males’ mean pension benefit) in the calculation of the actual GGP (see Table 5), and (ii) a slight increase in the absolute (money) gender gap from 4,709 to 4,813 euros, that is the numerator of the actual GGP.

Second, in Cell 3 the virtual GGP is found to be surprisingly lower than the actual GGP (0.201 and 0.202, respectively), meaning that females would have done slightly better relative to males under a fully NDC scheme. The motivation in this case is found in gender differences with respect to the starting working age and career discontinuities in early working life. Indeed, for the greatest part of the population in Cell 3, the DB scheme applies up to 1995, whereas the NDC scheme is in force for the rest of the working life. Since the starting working age is, on average, higher for females than for males (see Table 2) and since career discontinuities are more frequent in the early working life of females (about one year seniority gap in the early working life), the quota of years of contribution accrued in the DB scheme (i.e., before 1996) is, on average, higher for males than for females, implying that the generous DB schemes applies relatively more to males as compared to females in the same cell (Cell 3).^38^ Hence, due to the design of the pro-rata mechanism and to gender differences in the early contribution career, females are additionally penalized by a relatively stronger application of the NDC scheme with respect to males.

#### GE Decomposition

In this section, an alternative approach to the measurement of the gender gap at retirement is implemented, by which the size and the dynamics of both within-gender and overall inequality are observed in addition to between-gender inequality. With this purpose in mind, we calculate and decompose the GE inequality measures discussed above (Eq. 3). In Table 7, we only report results obtained under the hypothesis $\alpha =1$ in Eq. (2), which is known as the Theil inequality index; results for different values of the parameter $\alpha$ do not differ substantially from each other and are reported in Appendix A.5 (Table 11).

**Table 7:**
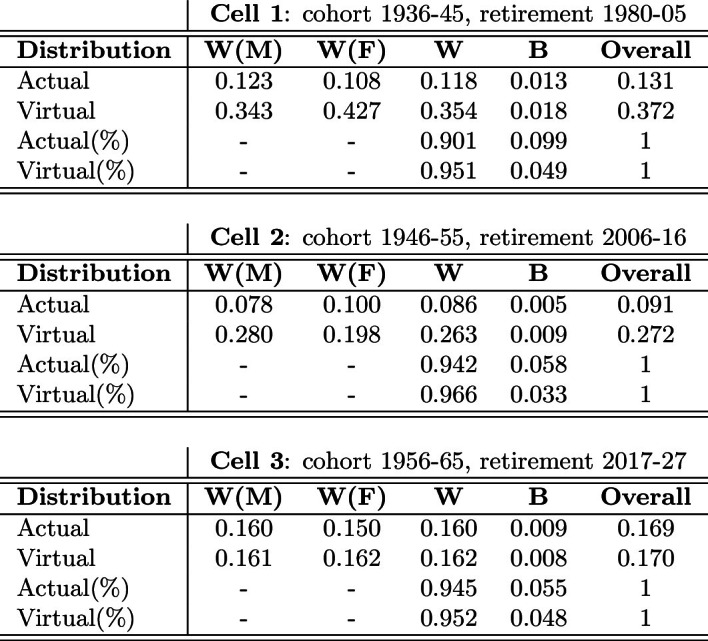
Actual and virtual subgroup inequality components (Theil index)

We first observe that all the results obtained from the GGP analysis are confirmed when decomposing the Theil inequality index. Specifically, by looking at the between-gender component (column B), the U-shape of the actual GGP across cells is confirmed here in that the actual between-gender inequality is found to decrease from Cell 1 to Cell 2 (from 0.013 to 0.005), while it increases from Cell 2 to Cell 3 (from 0.005 to 0.009). Second, the virtual between-gender inequality is found to be decreasing over time (from 0.018 in Cell 1 to 0.008 in Cell 3) due to improving labor market conditions of women, especially from Cell 1 to Cell 2. Third, in line with the GGP analysis, we find that the between-gender inequality in the virtual distribution of pension benefits is greater than in the actual one for both Cell 1 (0.018 vs 0.013) and Cell 2 (0.009 vs 0.005) but not for Cell 3 (0.008 vs 0.009), which proves the existence of perverse (poor-to-rich) redistribution in Cell 3.

Since the GE decomposition also captures the dynamics of within-gender (column W) and overall inequality (column Overall), further insights can be inferred concerning the nature of the perverse between-gender redistribution observed in Cell 3. Indeed, from Table 7, the DB pension system is found to reduce within-gender inequality, *W*, for both males (column *W*(*M*)) and females (column *W*(*F*)), as well as overall inequality. This is particularly evident in Cell 1, where the Theil inequality index for the virtual distribution (0.372) is more than twice the one calculated with respect to the actual distribution of pension benefits (0.131). As one may expect, due to the large application of the NDC scheme, this redistributive effect becomes tiny in Cell 3 (0.170 vs 0.169) but, for what it’s worth, it is still there. The existence of a rich-to-poor redistributive effect in Cell 3 with respect to the overall distributions of pension benefits proves that the perverse (poor-to-rich) between-gender redistribution in Cell 3 (column B) is not originating from generic perverse redistribution (column Overall), but it must be gender-specific in nature, that is women’s penalization in the pro-rata mechanism discussed above.^39^

Finally, since the GE decomposition also provides information on overall inequality among retirees in each cell, in Table 7 we also report the share (%) of actual and virtual between-gender inequality with respect to the overall inequality for each cell. It is worth observing that the share of between-gender inequality falls from Cell 1 to Cell 2 (from 9.9% to 5.8%) but remains almost stable in Cell 3 (5.5%). This result highlights that the share of gender inequality with respect to overall inequality is no longer expected to improve for the population of retirees in the near future.

## Concluding Remarks

The assessment of the recent trend in the evolution of the gender gap at retirement is particularly important in Italy because it allows us to gather key information on the impact of pension reforms adopted during the nineties and, eventually, to correct for undesirable tendencies observed nowadays.

To account for the interaction between pension reforms and evolving labor market conditions for women, a counterfactual analysis is implemented in this paper by which the gender gap at retirement is calculated—across cohorts and years of retirement—using both the actual and the virtual distribution of pension benefits with the latter being obtained under the hypothesis of a fully NDC scheme. Most importantly, virtual pension benefits are calculated by reconstructing the entire working career of individuals from real data (SHARELIFE, wave 3 and 7).

Given the actual and the virtual distributions of pension benefits, both the GGP analysis and the GE decomposition have been implemented. The two methodologies lead to similar results on the dynamics of the gender gap at retirement; the GE decomposition also highlights the loss of the redistributive power due to the transition from the DB to the NDC scheme, as well as the incidence of gender disparities on overall and within-gender disparities among retirees with an old-age or seniority pension.

We find a U-shaped behavior of the actual gender gap at retirement in Italy, with an ascendant pattern starting from 2017. Hence, we conclude that, while the improvement of labor market conditions for women has been dominating up to 2016 to reduce the gender gap at retirement, this effect is expected to be dominated by the loss of redistributive power of the pension scheme after this date. Even worst, the share of gender inequalities with respect to overall inequality among retirees is found almost stable in recent times, meaning that gender disparities are still important among all other determinants of inequality.

We also show that the application of the pro-rata mechanism during the transition from the DB to the NDC scheme is additionally penalizing for women in that, on average, (i) the higher starting working age and (ii) career discontinuities in early working life reduce, especially for women, the quota of pension savings falling in the more generous DB scheme. This effect is limited but not negligible since it makes the actual gender gap at retirement observed after 2016 greater than what this would have been if a fully NDC scheme were applied.

Altogether, in the transition from the DB to the NDC scheme, women turn out to be sensibly penalized by both the loss of redistributive power of the pension plan, as well as by lower pension savings in the early working life with respect to men. As such, our analysis suggests that gender-specific redistributive policies, especially those related to family caring in the early working life, are urgently required in Italy to arrest the increasing trend of the gender gap at retirement observed in recent times.

## Figures and Tables

**Table 8:**
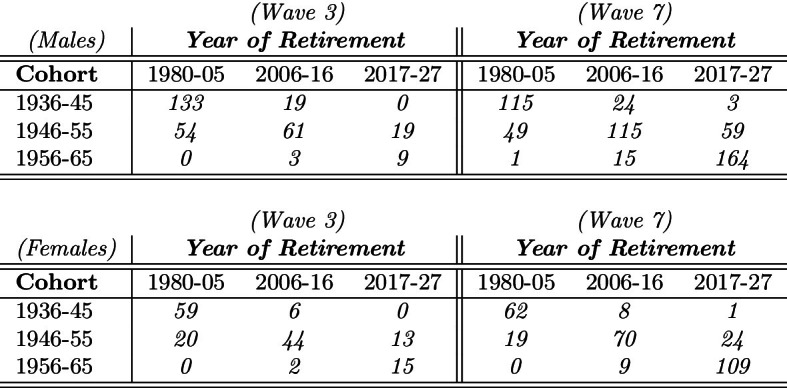
Number of observations by cohort, year of retirement, gender, and wave

**Table 9:**
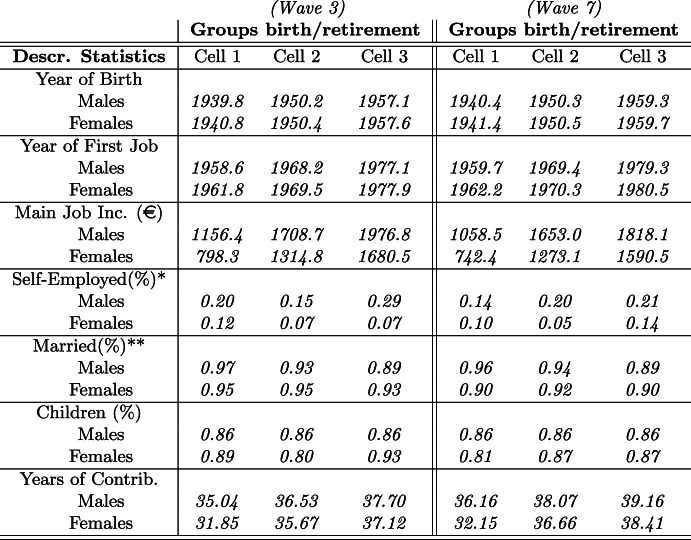
Descriptive statistics by group, gender, and wave

$^*$    If self-employed and civil servant for one employment spell at least

$$^{**}$$ If married at least once

**Table 10:**
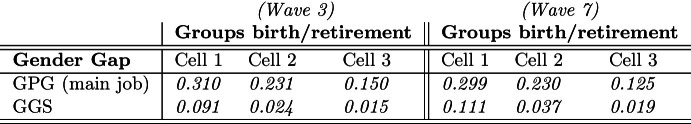
Gender pay gap (main job) and gender gap in seniority by wave

**Table 11:**
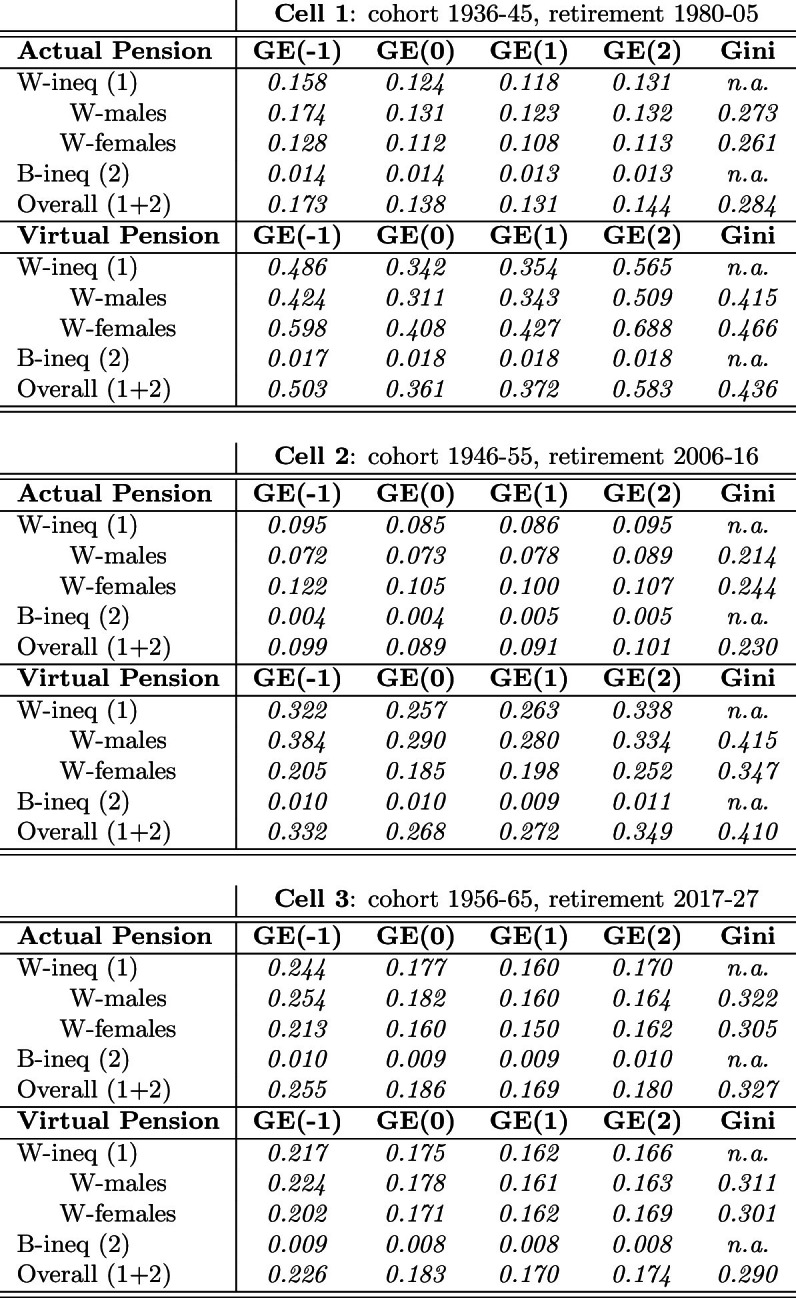
Inequality decomposition

## Appendix

**A.1 Original Sample**

**A.2 Descriptive Statistics**

**A.3 Gender Gaps**

**A.4 Transformation Coefficients: Definition and Estimation**

*Definition* According to the Italian Legislation, Transformation Coefficients are used to transform in pension the contributions accrued until the retirement age. Let $P_x=(TC_x)(MC_x)$ be the annual pension benefit for a worker retiring at age *x*, where $MC_x$ is the total amount of accrued contributions at retirement age *x*, and $TC_x=\frac{1}{\Delta _x}$ is the Transformation Coefficient at retirement age *x* with

$$\begin{aligned} \Delta _x= \frac{\sum ^{}_{s=m,f}\left( a^{v(t)}_{x,s}+A^{v(t)}_{x,s}\right) }{2}-k \end{aligned}$$

known as the Divisor. The Divisor, making use of actuarial symbology, involves both the average present value of the individual’s pension, $a^{v(t)}_{x,s}$, and the average present value of the survivor’s pension of both men and women, $A^{v(t)}_{x,s}$, where:

$$\begin{aligned} {a^{v(t)}_{x,s}}={\sum ^{w-x}_{t=0}}\frac{l_{x+t,s}}{l_{x,s}}\left( \frac{1+r}{1+\sigma }\right) ^{-t} \end{aligned}$$

and

$$\begin{aligned} {A^{v(t)}_{x,s}}={\sum ^{w-x}_{t=0}}\frac{l_{x+t,s}}{l_{x,s}}q_{x+t,s}\left( \frac{1+r}{1+\sigma }\right) ^{-t}\Theta _{x+t,s}\eta \delta _s\sum ^{w-x-t+\varepsilon _s}_{\tau =1}\frac{l^{ved}_{x+t+\tau -\varepsilon _{x,s},\bar{s}}}{l^{ved}_{x+t+1-\varepsilon _{x,s},\bar{s}}}\left( \frac{1+r}{1+\sigma }\right) ^{-\tau } \end{aligned}$$

*w* = ultimate age *s* = sex (m = male; f = female) $\bar{s}$ = sex of the surviving spouse $\frac{l_{x+t,s}}{l_{x,s}}$ = survival probability between age *x* and $x+t$ $q_{x+t,s}$ = probability of death between age *x* and $x+t$ $\Theta _{x+t,s}$ = probability to leave the family for a person aged $x+t$ $l^{ved}_{x+t,s}$ = probability of the survivor to die or remarry $\varepsilon _{x,s}$ = age gap between retiree and spouse $\eta$ = survivor benefit rate (fixed at 0.6) $\delta _s$ = income-based means-tested percentage reduction of the survivor benefit rate (fixed at 0.9 for males, 0.7 for females) *r* = internal rate of return $\sigma$ = indexing factor $\left( \frac{1+r}{1+\sigma }\right) ^{-\tau }$ = discount rate (with $\left( \frac{1+r}{1+\sigma }\right)$ fixed at 1.015) *k* = correction factor for monthly payments in advance during the year (fixed at 0.4615).

$TC_x$ (or better its reciprocal, i.e. the Divisor) is expected to ensure the equivalence between the amount of accrued contributions and the annuity payed to the retiree until s/he or her/his survivors die (depending on life expectancy).

*Estimation strategy* Currently, official $TC_x$s from ISTAT are available until 2022. To update the $TC_x$s from 2023 ahead, we project survival probabilities at the year of retirement of the retirees—ranging from 57 up to 71 years old according to the Italian legislation—throughout the Lee–Carter model (Lee and Carter 1992) characterized by the following equation:

$$\begin{aligned}\ln m_{x,t}=\alpha _x+\beta _x\kappa _t+\varepsilon _{x,t} \end{aligned}$$

where $\ln m_{x,t}$ is the logarithm of the observed mortality rate $m_{x,t}$ for age *x* and year *t*. On the right hand side of the equation, $\alpha _x$ is an age-specific and time-independent component, while the second component is given by the product of a time-varying parameter $\kappa _t$, reflecting the general level of mortality, and $\beta _x$, an age-specific component indicating how mortality at each age varies when the general level of mortality changes. $\varepsilon _{x,t}$ designates the error term, which is assumed to be homoschedastic and normally distributed.

As the projected survival probabilities at the extreme age undertake irrelevant values, we assume that the ultimate survival probabilities (from 103 up to 119 years old) are set equal to the expected survival probability at the selected ultimate age (102 years old).

The steps for forecasting $m_{x,t}$ are summarized as follows.

1. The basic Lee–Carter model is fitted to the selected dataset. Using the Singular Value Decomposition (SVD) method, assuming suitable normality constraints in order to obtain a unique solution, the model is fitted to a matrix of age-specific observed mortality rates.
2. The model parameters $\alpha _{x}$, $\beta _{x}$ and $\kappa _{t}$ are estimated, and to obtain a unique solution, we impose that the sum of the $\beta _{x}$ coefficients is equal to one, and the sum of the $\kappa _{t}$ parameters is equal to zero.
3. Next, the mortality index $\kappa _{t}$ are forecasted by using ARIMA models.
4. Finally, forecasted mortality rates for 13 years ahead from 2018 to 2030 and survival and death probabilities are derived.

It has to be highlighted that the $TC_x$s have been updated by taking into account not only the changes occurred in the probability of death, but also other parameters provided by ISTAT. Specifically, we use ISTAT data on the projected probabilities of death or remarriage of the surviving spouse; the average age differential between spouses at death of partner; the probability of leaving the family, which is inferred by ISTAT from the relative frequency distribution of deaths by age in completed years, sex, and marital status. For the purposes of our analysis, we set $t = 2022$, 2024, 2026 as baseline years, respectively, for the $TC_x$s in bienniums 2023–2024, 2025–2026, and 2027–2028.

**A.5 Decomposition of Generalized Entropy Measures**

## Footnotes

1 An actuarially fair scheme guarantees that, for each individual, the discounted amount of contributions paid during the working career is equal to the discounted amount of expected benefits received during retirement. For major details see Belloni and Maccheroni (2013).

2 We strictly focus on the first pension benefit received after retirement. In a lifecycle perspective, instead, different indicators (net present-value ratio, internal rate of return, social security wealth) may be required in order to properly account for the dynamics of pension benefits after retirement (Belloni et al. 2020, Mazzaferro 2019).

3 Even if adequate pension systems are expected to supply both insurance and social assistance (Abatemarco 2009), it is often the case that a distinction is made between these two services. For major details on the interaction between insurance and social assistance targets in public pension plans, see Diamond (2004).

4 For the purposes of our analysis, as well as for the lack of sufficient information, we do not consider minor pension reforms, whose impact on the population of retirees is marginal and mostly limited to specific work categories.

5 For details on the Italian reform process, see Coda Moscarola and Fornero (2009), Belloni et al. (2013), Brugiavini and Galasso (2004), Giuliani (2020).

6 The number of years to be included in the computation of pensionable earnings has been progressively increased by reforms in the nineties from 5 up to 10 for employees and from 10 up to 15 for self-employed workers.

7 Even if the benefit formula of the Italian DB scheme is redistributive (Mazzaferro 2019), evidence of perverse redistribution has been reported in some cases (Abatemarco 2006, Borella 2004, Castellino 1995, Borella and Coda Moscarola 2006, Fornero and Castellino 2001).

8 This reform also changed the indexation mechanism of the first pension by eliminating the adjustment to the dynamics of real wages, while preserving the link to price inflation.

9 The accrual rate for “Quota B” is set at 2% for pensionable earnings below a reference level legislated annually (inflation-adjusted), at 1.60% if pensionable earnings are between the reference level and 1.33 times the reference level, at 1.35% between 1.33 and 1.66 times the reference level, at 1.10% above 1.66 times the reference level, and for the highest earnings bracket (now consisting of earnings exceeding 1.90 times the reference level) was lowered from 1 to 0.90%.

10 The Transformation Coefficients vary from 57 to 71 years old according to the worker’s retirement age.

11 The decision to safeguard the interests of elder workers by preserving a very generous DB formula was mostly driven by political motivations.

12 After the Dini reform, several acts intervened to adopt more stringent rules with respect to both eligibility requirements and pensionable earnings. In 1997, to reduce once more the tax burden of the pension system, the Prodi government raised the retirement age, which was additionally increased a few years later by the Berlusconi government (Law 243/2004). A further step was taken in 2007, when the Prodi-Damiano Reform (Law 247/2007) modified the previous reform by introducing the use of quotas, i.e., age and contribution requirements for claiming pension, whose aim consisted of progressively increasing the retirement age. As for the retirement age, the Sacconi reform (Law 102/2009) introduced for the first time an indexing mechanism by which the retirement age is automatically adjusted for changes in life expectancy.

13 For workers achieving this threshold before 62 years old, a penalization with respect to the pre-2012 quota of the pension benefit applies.

14 The largest gaps were recorded in Malta (46.1%), the Netherlands (43.4%), Luxembourg (42.6%), Austria (41.1%) and Cyprus (41.1%).

15 In the U.S., where social security is not intended to be the primary source of retirement income for men or women, the average social security retirement benefit in 2020 was $16,536 for women, compared with $20,568 for men, with a roughly 20% GGP (SSA 2021).

16 The SHARE data collection has been funded by the European Commission through FP5 (QLK6-CT-2001-00360), FP6 (SHARE-I3: RII-CT-2006-062193, COMPARE: CIT5-CT-2005-028857, SHARELIFE: CIT4-CT-2006-028812), FP7 (SHARE-PREP: GA N.211909, SHARE-LEAP: GA N.227822, SHARE M4: GA N.261982, DASISH: GA N-283646) and Horizon 2020 (SHARE-DEV3: GA N.676536, SHARE-COHESION: GA N.870628, SERISS: GA N.654221, SSHOC: GA N.823782) and by DG Employment, Social Affairs and Inclusion. Additional funding from the German Ministry of Education and Research, the Max Planck Society for the Advancement of Science, the U.S. National Institute on Aging (U01_AG09740-13S2, P01_AG005842, P01_AG08291, P30_AG12815, R21_AG025169, Y1-AG-4553-01, IAG_BSR06-11, OGHA_04-064, HHSN271201300071C) and from various national funding sources is gratefully acknowledged (see http://www.share-project.org).

17 The database exceptionally includes individuals younger than 50 when married/living with respondents aged 50 years old at least.

18 In wave 7, SHARELIFE modules were asked to all respondents who did not participate in wave 3.

19 We thank two anonymous referees for suggesting us to join the two waves in order to improve the robustness of our results.

20 Alternatively, the EU-SILC database may be considered for similar research purposes. Even if SHARE and EU-SILC differ from each other for the income definition and available information, it has been observed that these differences are not sufficient to jeopardize the compatibility of the ‘big picture’ emerging from the two databases (Tinios et al. 2015).

21 Methodological details are provided in the next section.

22 Unfortunately, descriptive statistics on education are omitted due to excessive number of missing values.

23 Notably, the GGS is expected to be greater when considering first-tier pensions which have been excluded from this analysis due to the focus on the sole insurance mechanism.

24 The application of *unisex* Transformation Coefficients in the NDC scheme may implicitly cause redistribution due to gender differentials of mortality rates (Leombruni and Mosca 2012). However, this redistribution has been found lower in recent official estimates. For example, in the 2019 update of the official Transformation Coefficients for individuals retiring at the age of 67 years (or more), the unisex Transformation Coefficient is penalizing females due to a dominating survival component (see Appendix A.4).

25 This is particularly relevant if considering the impact of gender-specific redistributive policies (e.g., fictitious contribution for maternity leave or caregiving) as opposed to generic rich-to-poor redistributive policies.

26 The salary growth rate for each year is obtained from yearly variations in gross hourly earnings in main economic sectors from ISTAT’ report “Numeri indice delle retribuzioni contrattuali orarie lorde per alcuni settori di attività economica e qualifica professionale—Anni 1955–2015”. These data are not disaggregated by gender, even if a statistically significant gender gap in salary progression may be there for some subgroups of the population (e.g., Barth et al. 2021).

27 Mainly, this excludes shareholder’s profits, capital gains, cadastral and real estate incomes, whose impact however is expected to be small, in that the greatest part of capital incomes (interests from bonds, bank accounts and deposits) and real estate incomes (rents) are source taxed according to the Italian legislation, and so excluded from the personal income taxation.

28 Before 1973, the Italian tax system was sensibly different from modern tax systems, and mostly based on withholding and indirect taxation.

29 For details on the evolution of the Italian personal income tax, see OECD (2005) and Pellegrino and Panteghini (2020).

30 According to the Italian pension system, 1/3 of the overall pension savings accrued for the worker must be paid by the worker itself, whereas 2/3 are paid by the employer.

31 The contribution rate for self-employment is not uniquely defined for different types of self-employed workers and has been sensibly modified in the last decades (mostly increased). Here, we use the 20% as a proxy for all the different categories which we cannot disentangle from the information available in the SHARE database.

32 Due to lack of information, we do not consider departures from the main eligibility criteria applied to specific work categories (e.g., special treatment for heavy occupations). In addition, we do not consider the so called “Opzione Donna” (first introduced by Law 243/2004), by which females with specific age and seniority requirements may opt for early retirement by accepting the application of the NDC scheme for the entire contribution career.

33 Notably, we also preserve the unemployment status for unemployed (and non-retired) respondents at the time of the interview; however, the greatest part of these observations are dropped due to unmet eligibility requirements for an old-age or seniority pensions within 2027.

34 We consider hourly earnings growth rate from ISTAT’s “Indici delle retribuzioni contrattuali orarie per raggruppamenti principali di contratto” until September 2020 differentiated by macro sectors (agriculture, industry, public administration, general index).

35 Notice that mortality projections do not account for the impact of COVID-19 since data provided by the HMD are updated to 2019. However, to the extent that Transformation Coefficients are uniformly applied to males and females according to current Italian legislation, COVID-19 is not expected to sensibly affect the gender gap.

36 The GDP grow rate at market prices (chained values; reference year = 2010) from 1950 to 2017 is extracted from “La Ricostruzione Banca d’italia—Istat 1861–2017” available from the official website of the ISTAT.

37 Since the final dataset consists of a small subsample of all observations in SHARE, and since the subsample is obtained by merging two different waves (3 and 7), sample weights are ignored in our computations. Also, since we are focused on the sole insurance mechanism of pension schemes, we decided to keep observations with very low pension benefits, which are associated to missing social integration to a minimum due to the presence of other incomes within the household.

38 More precisely, this occurs for the greatest part of the population in Cell 3 which is expected to become eligible first for an old-age (not seniority) pension.

39 Also notice that, in the transition from Cell 1 to Cell 2 (Table 7), actual within-gender inequality increases whereas virtual within-gender inequality moves in the opposite direction. This result is driven by the significant lack of actuarial fairness of the DB scheme (Cell 1), which is captured by the substantial gap between virtual within-gender inequality (reflecting inequality in lifetime earnings) and actual within-gender inequality (reflecting inequality in pensionable earnings and years of contribution).
